# Optimizing Human Bile Preparation for Two-Dimensional Gel Electrophoresis

**DOI:** 10.1155/2016/5185317

**Published:** 2016-02-04

**Authors:** Hao-Tsai Cheng, Sen-Yung Hsieh, Chang-Mu Sung, Betty Chien-Jung Pai, Nai-Jen Liu, Carl PC Chen

**Affiliations:** ^1^Division of Gastroenterology, Department of Internal Medicine, Linkou Chang Gung Memorial Hospital and Chang Gung University College of Medicine, Taoyuan, Taiwan; ^2^Graduate Institute of Clinical Medicine, College of Medicine, Chang Gung University, Taoyuan, Taiwan; ^3^Department of Gastroenterology and Hepatology, Chang Gung Memorial Hospital and Chang Gung University College of Medicine, Taoyuan, Taiwan; ^4^Department of Craniofacial Orthodontics, Chang Gung Memorial Hospital, Taipei, Taiwan; ^5^Graduate Institute of Craniofacial and Oral Science, Chang Gung University, Taoyuan, Taiwan; ^6^Craniofacial Research Center, Chang Gung Memorial Hospital, Linkou, Taiwan; ^7^Department of Physical Medicine & Rehabilitation, Chang Gung Memorial Hospital at Linkou and College of Medicine, Chang Gung University, Kwei-Shan, Taoyuan, Taiwan

## Abstract

*Aims*. Bile is an important body fluid which assists in the digestion of fat and excretion of endogenous and exogenous compounds. In the present study, an improved sample preparation for human bile was established.* Methods and Material*. The method involved acetone precipitation followed by protein extraction using commercially available 2D Clean-Up kit. The effectiveness was evaluated by 2-dimensional electrophoresis (2DE) profiling quality, including number of protein spots and spot distribution.* Results*. The total protein of bile fluid in benign biliary disorders was 0.797 ± 0.465 *μ*g/*μ*L. The sample preparation method using acetone precipitation first followed by 2D Clean-Up kit protein extraction resulted in better quality of 2DE gel images in terms of resolution as compared with other sample preparation methods. Using this protocol, we obtained approximately 558 protein spots on the gel images and with better protein spots presentation of haptoglobin, serum albumin, serotransferrin, and transthyretin.* Conclusions*. Protein samples of bile prepared using acetone precipitation followed by 2D Clean-Up kit exhibited high protein resolution and significant protein profile. This optimized protein preparation protocol can effectively concentrate bile proteins, remove abundant proteins and debris, and yield clear presentation of nonabundant proteins and its isoforms on 2-dimensional electrophoresis gel images.

## 1. Background

Bile is an important body fluid which assists in the emulsification and adsorption of fat and functions as a vehicle for the excretion of bilirubin, metabolites, toxins, phospholipids, and inorganic ions [1]. Bile is produced by hepatocytes and stored in gallbladder with concentrated hepatic bile [2]. Biliary tract diseases include cholecystitis, cholelithiasis, and malignancies of the biliary tract system [3]. Accurate diagnostic approaches to interpret the changes in biomarkers related to biliary diseases are in great demand [2].

Proteomic approaches have been used for clinical biomarker discovery in a variety of tissues and body fluids including cerebrospinal fluid (CSF), synovial fluid (SF), and plasma [4]. Two-dimensional gel electrophoresis (2DE) is a common approach for proteomic analysis, which separates and identifies hundreds of proteins simultaneously [5]. Serum and plasma are the sample of choice for biomarker studies as they can be easily retrieved [6]. However, biomarker concentrations of body fluids may present significant differences, giving up to 12 orders of magnitude [7].

For the monitoring of biliary disorders, a baseline proteome profile of bile is necessary for the discovery of potential biomarkers associated with specific biliary diseases. Proteomics has been applied to discover the biomarkers that may be associated with diagnosis, prognosis, and treatment of biliary diseases [8]. Disease related biomarkers usually appear in low concentrations and may be masked by high abundant proteins [9]. Removal of uninformative high abundant proteins is suggested to enhance the sensitivity of disease related proteins. However, some studies have demonstrated that such removal could affect the concentrations of proteins that may be of interest to us [9]. In this study, sample preparation methods of acetone precipitation and 2DE Clean-Up kit were examined for their effectiveness on the enhancement of proteome profile of human bile.

Acetone precipitation method has been reported to improve proteome profile of body fluids with low protein concentration and high salt content such as CSF [10]. Bile composes water (85%), salts (10%), mucus and pigments (3%), fats (1%), and inorganic salts (0.7%). Total protein concentration of bile is expected to be low. The high salt content of bile is expected to have a tremendous influence on resolution of 2DE analysis.

In this study, acetone precipitation, 2DE Clean-Up kit, and a combination of these two methods were investigated on their influences on bile protein presentation on 2DE gel images. We hypothesized that treating bile samples with acetone first followed by 2DE Clean-Up kit can yield the best results in the presentation of bile proteins on 2DE gel images.

## 2. Materials and Methods

### 2.1. Subjects and Sample Gathering

Bile fluids were collected during endoscopic retrograde cholangiography (ERCP) from patients with biliary stenosis to different benign diseases. Benign diseases were biliary pancreatitis, common bile stones, biliary tree infection, or chronic pancreatitis. Over 10 mL of bile was collected, aliquoted, and stored at −20°C. The aliquots were used for experimental analysis within one week. Samples were stored at −80°C for long-term storage.

A total of 12 patients with benign biliary diseases were recruited. Their samples were used for the calculation of bile fluid total protein concentrations. All patients signed the informed consent before participating in this study. The Chang Gung Memorial Hospital institutional ethics committee approved all the protocols involved in this study.

### 2.2. The Calculation of Bile Fluid Total Protein Concentrations

The Bradford method was applied for the calculation of protein concentrations [11]. A standard curve was developed using bovine serum albumin (BSA) (Sigma, USA) at serial concentrations (2, 1, 0.5, 0.25, and 0.125 *μ*g). The BioRad Protein Assay dye reagent was used. The dye-proteins were measured by Unicam UV1 spectrophotometer at an absorbance of 595 nm.

### 2.3. Two-Dimensional Electrophoresis (2DE)

Isoelectric focusing (IEF) was performed using 17 cm, pI 3–10 nonlinear IPG strips. The rehydration buffer containing the untreated bile fluid or protein precipitate (after treatment with acetone, 2D Clean-Up kit, or the combination of both) was loaded onto the IPG strips and rehydrated overnight (in-gel rehydration method) at room temperature. The strips were focused at 50 *μ*A/IPG strip for a total of 60 kVh at 20°C using the Protean IEF Cell (BioRad). After focusing, strips were equilibrated with 1% DTT and 4.8% iodoacetamide for 15 min. After equilibration, IPG strips were washed with SDS-polyacrylamide (SDS-PAGE) running buffer and then applied onto the top of 12% SDS-PAGE gels. The second dimension separation in the second dimension was carried out using the Protean II electrophoresis equipment and Tris-glycine buffer (25 mM Tris, 192 mM glycine) containing 0.1% SDS. The running condition was set at constant 40 mA per gel and with the temperature at 10°C. Gels were placed in a fixing solution (containing 50% methanol and 7% acetic acid) overnight in preparation for protein visualization using SYPRO Ruby Protein Gel Stain (Molecular Probes, Invitrogen).

### 2.4. Image Analysis

The ProXPRESS™ 2D Proteomic Image System (PerkinElmer Life and Analytical Sciences) was used to scan the SYPRO Ruby stained SF 2DE gels. The scanner was set with an excitation of 520 nm and emission filter of 610BP30 as recommended by the product protocol. The gel images were acquired as digital TIF files and analyzed using the PDQuest Basic 8.0.1 Analytical software (BioRad).

### 2.5. Protein Identification by Mass Spectrometry (MS)

Protein spots of interest were manually excised from the SYPRO Ruby stained SF 2DE gels and destained using 50 mM NH4HCO3 in 50% ACN and dried in a SpeedVac concentrator. The protein was then digested by incubating overnight at 37°C with trypsin (Promega, Madison, WI; at 5 ng/mL) in 50 mM NH4HCO3, pH 7.8. Tryptic peptides were extracted from the gel pieces in 1 volume of 0.1% TFA, while vortexing for 5 minutes, followed by sonication for 5 minutes. Crude digest mixtures were concentrated and desalted using mC18 Zip-Tips (Millipore) followed by eluting in 1.5 mL of matrix (5 mg of CHCA/mL in 50% ACN/0.1% TFA) for MALDI-TOF MS and MS/MS analyses. Both MS and MS/MS spectra were searched against the NCBI database, using MASCOT software from matrix science (http://www.matrixscience.com/), to identify the proteins. The MALDI-TOF MS resolution for the peptides was around 20,000, and the mass accuracy was 0.01–0.02 Da.

### 2.6. Data Analysis

The numbers of protein spots was detected by the PDQuest Basic 8.0.1 Analytical software from the constructed 12 2DE gel images. The bile fluid protein concentrations, numbers of protein spots from untreated bile fluid samples, and samples treated with acetone and/or 2D Clean-Up kit were expressed as mean ± standard error of means (SEM). ANOVA and* t*-test were used as appropriate to compare the means from different bile fluid sample treatments. The Statistical Program for Social Sciences (SPSS) version 13 (SPSS Inc., Chicago) was used for data calculations. Values of *p* < 0.05 were considered statistically significant.

## 3. Results

**Figure 1:**
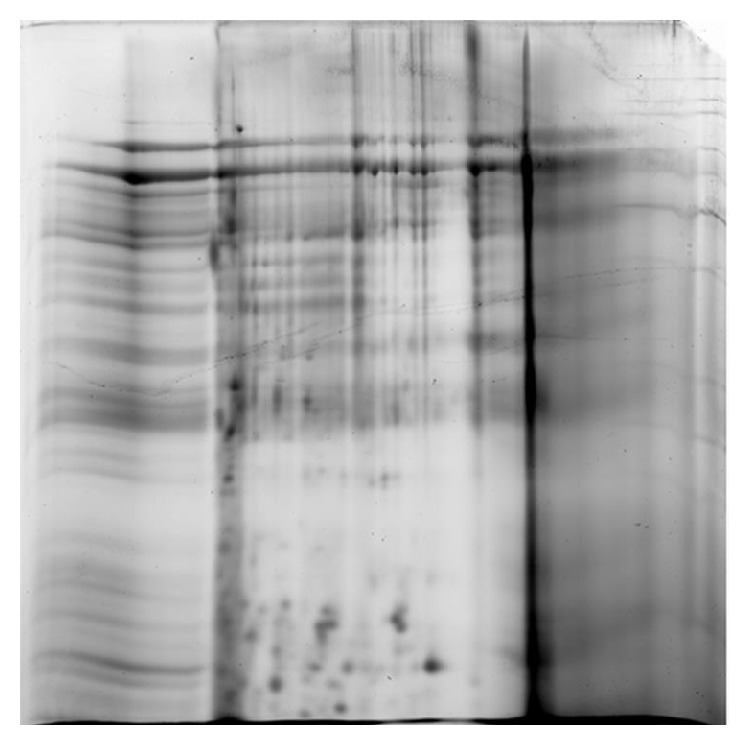
Two-DE gel image of untreated bile sample was detected to have approximately 39 protein spots.

**Figure 2:**
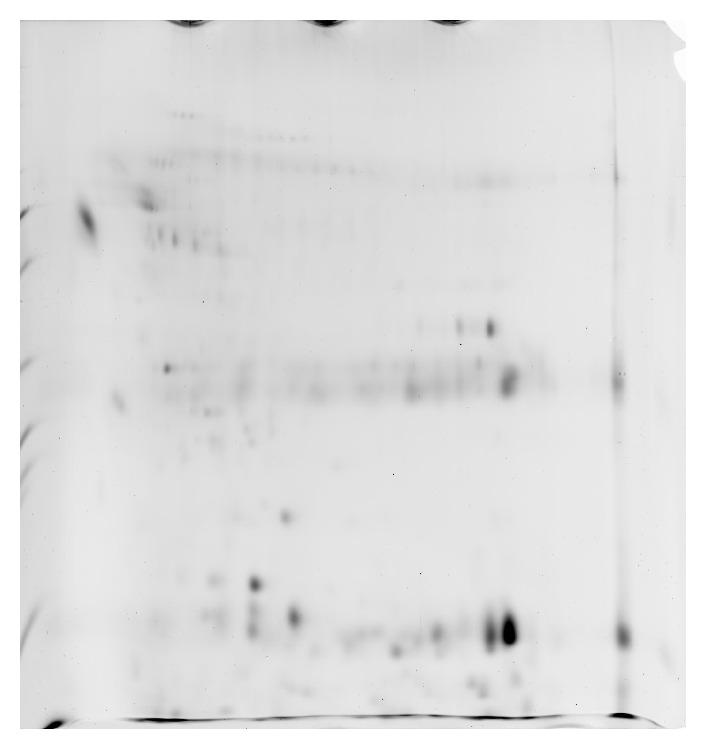
Two-DE gel image of bile sample treated with 2D Clean-Up kit was detected to have approximately 52 protein spots.

**Figure 3:**
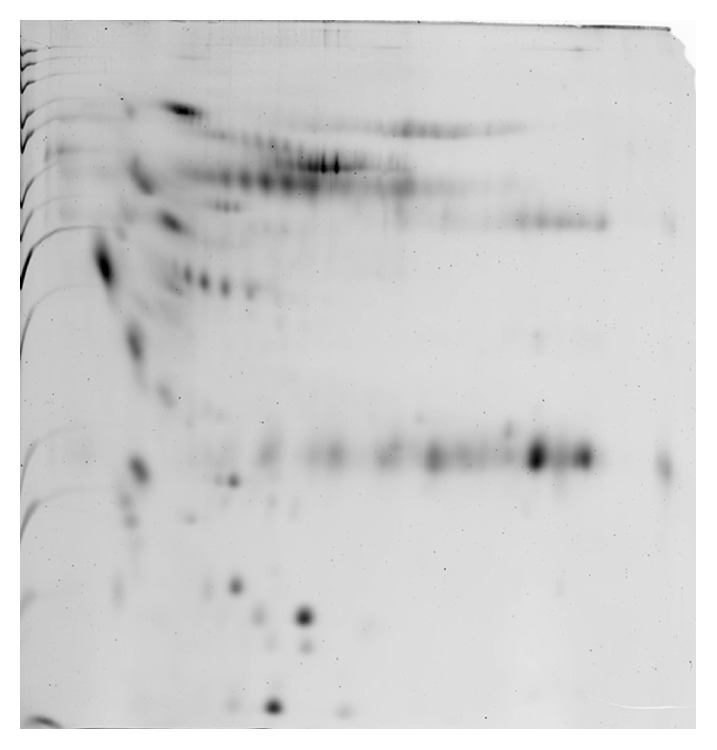
Two-DE gel image of bile sample treated with acetone precipitation method was detected to have approximately 170 protein spots.

**Figure 4:**
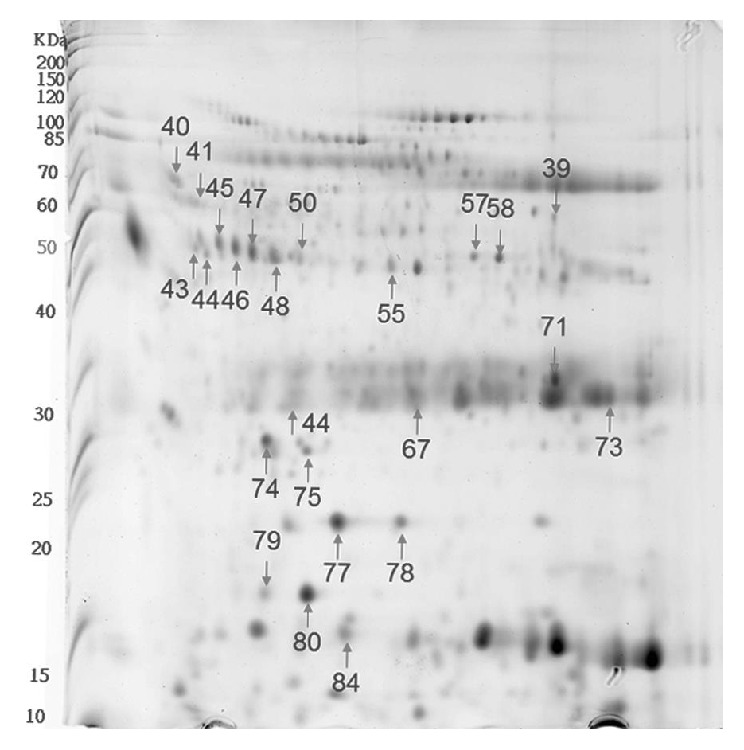
Two-DE gel image of bile sample treated with acetone first and then subsequently by 2D Clean-Up kit. This gel image was detected to have approximately 558 protein spots.

The total protein concentration of human bile for the benign biliary disorders was 0.797 ± 0.465 *μ*g/*μ*L. The untreated bile sample was examined using 2DE, resulting in approximately 39 protein spots (Figure 1). Sample preparation using 2D Clean-Up kit led to the discovery of a 52-protein spots and a concentration of 0.598 ± 0.213 *μ*g/*μ*L (Figure 2). Bile sample treated with acetone precipitation method exhibited a profile of 170 protein spots that was significantly higher than that of untreated sample and a concentration of 1.478 ± 0.415 *μ*g/*μ*L (Figure 3). Protein preparation using acetone followed by 2D Clean-Up kit resulted in a significant increase in number of protein spots on 2DE compared to those of the other two methods, giving approximately 558 spots and a protein concentration of 0.866 ± 0.331 *μ*g/*μ*L (*p* < 0.05, Figure 4). Tables 1 and 2 showed the data of bile fluid protein spot numbers and total concentrations after different treatment methods.

The protein spots discovered which were confirmed by MS and analyzed subsequently by the SWISS-PROT database are listed in Table 3. As shown in Figure 4, bile fluid preparation using acetone followed by 2D Clean-Up kit method developed in this study led to a better resolution of 2DE gel images. The identified protein spots appeared to have more clear protein spots of haptoglobin, serum albumin, serotransferrin, and transthyretin as compared with untreated samples and samples treated with 2D Clean-Up kit and acetone alone. Images of 2DE analysis revealed that preparation using acetone followed by 2D Clean-Up kit successfully removed the abundant proteins such as albumin and increased sensitivity to nonabundant proteins on 2DE gel images.

## 4. Discussion

Bile fluid is clinically obtained through endoscopic retrograde cholangiography (ERCP) [12]. Proteome analysis of bile fluid from benign liver diseases requires a reference 2DE gel image that can be used for comparison to discover protein spots (biomarkers) that are associated with nonbenign liver diseases. In the present study, we developed a suitable sample treatment method that can enhance the presentation of less abundant protein spots and their isoforms on 2DE gels can be beneficial for research on biliary disorders.

We compared the influences of acetone and 2D Clean-Up kit, and the combination of both sample treatment methods on bile fluid 2DE gel protein presentations. The SYPRO Ruby staining method was chosen due to its good linearity for data comparison [13]. Albumin and immunoglobulin G constitute about 65% and 15% of total protein concentrations in the plasma, respectively. The identification of potential less abundant disease biomarkers may be hindered by these highly abundant proteins [9]. It is suggested that an efficient method that can remove these abundant proteins and not influencing the presentation of less abundant proteins on 2DE gel images is important in disease biomarker research.

There are numerous commercially kits and spin columns available to remove abundant proteins from samples [14]. The 2D Clean-Up kit used in this study has been demonstrated with efficient protein recoveries and the quality of 2DE gel images. It is a theoretically suitable kit for the preparation of bile fluid for 2DE analysis as bile fluid samples are rich in salt content and low in protein content and are often dirty with debris.

In this study, bile fluid samples treated with 2D Clean-Up kit yielded clean 2DE gel images, suggesting that 2D Clean-Up kit is effective in removing the debris, salts, and the unwanted precipitants in the bile fluid samples to ensure a clean 2DE gel image. However, the numbers of bile fluid protein spots on the 2DE gel images were still limited and revealed only 52 spots. The acetone precipitation method has been shown to have high protein recovery percentage and enhance the presentation of protein spots on 2DE gel images when used in treating body fluids with low protein concentrations and high salt content such as CSF [10]. It is implied to be a suitable sample treatment option for bile fluid as it has high salt content and low protein concentration. Acetone is suggested to wash away the salt content, as well as concentrating the bile fluid proteins for effective 2DE analysis. However, acetone fails to eliminate the highly abundant proteins as shown by the presence of albumin lump and heavy chain immunoglobulin G on 2DE gel images (Figure 3). The number of bile fluid protein spots on the 2DE gel images after acetone precipitation treatment method is still low, with only about 170 spots.

Our results show that it may be feasible to concentrate the bile fluid proteins first using the acetone precipitation treatment method followed by 2D Clean-Up kit. By using the combination of these two sample preparation methods, clear 2DE gel images with numerous protein spots of up to 558 spots were observed (Figure 4). The abundant proteins of albumin and immunoglobulin G were effectively removed. Nonabundant protein spots such as haptoglobin, serotransferrin, and transthyretin were clearly presented on the 2DE gel images. The number of albumin isoforms on the 2DE gel images also increased.

The same sample treatment method was previously used for knee SF samples but revealed different results [4]. The protein spots and concentrations actually decreased after using the same treatment method. This is because synovial fluids are rich in protein concentrations. Using acetone to increase protein recovery percentage and enhancing protein spot presentation on 2DE gel images is not needed for SF samples. Unlike CSF and bile fluid samples, acetone precipitation method will not increase protein concentration in synovial fluids [4]. Subsequent usage of 2D Clean-Up kit after acetone precipitation will then decrease the total protein concentration in SF samples.

In this study, 2DE gel images were developed for bile fluid samples. Good reproducibility was observed in all the 2DE gel images (>90% gel matching rate, data not presented). It is indicated that other liver disease proteomic studies can compare their gel images with the reference proteome pictures obtained in this study to assist them in differentiating whether the increase or decrease in certain protein spot intensities are disease related. For instance, an increase in the intensity of transthyretin protein spot of greater than 10% after bile fluid sample treatment with acetone and then 2D Clean-Up kit may indicate that transthyretin is a potential biomarker associated with that specific liver disease.

## 5. Conclusion

In conclusion, acetone precipitation method followed by 2D Clean-Up kit can effectively concentrate bile proteins, remove abundant proteins and debris, and yield clear presentation of nonabundant proteins and their isoforms on 2DE gel images. It is highly recommended that proteomic studies on bile fluid from liver diseases should be treated using acetone first and subsequently by 2D Clean-Up kit. It is crucial that a bile fluid 2DE gel image of benign liver disease shall be developed and used as a reference gel for comparative purposes with 2DE gel images derived from other liver diseases before drawing any conclusions on disease related biomarkers.

## Figures and Tables

**Table 1:** Number of detected protein spots on the 2DE gel images.

Bile fluid treatment method (*n* = 12)	Number of protein spots
Untreated	39
2D Clean-Up kit	52
Acetone precipitation method	170
Acetone followed by 2D Clean-Up kit	558^#^

^#^Comparison between acetone followed by 2D Clean-Up kit and the other 3 treatment methods (*p* < 0.05).

**Table 2:** Comparisons of total protein concentrations after different bile fluid sample treatment methods.

Bile fluid treatment method (*n* = 12)	Concentration in *μ*g/*μ*L
Untreated	0.797 ± 0.465
2D Clean-Up kit	0.598 ± 0.213
Acetone precipitation method	1.478 ± 0.415^#^
Acetone followed by 2D Clean-Up kit	0.866 ± 0.331

^#^Comparison between acetone followed by 2D Clean-Up kit and the other 3 treatment methods (*p* < 0.05).

**Table 3:** Names of the protein spots identified in this study confirmed by mass spectrometry (MS).

Sample name	Score	Spot name	Description	Acces. number	Mr (kDa)	PI	Number of matched peptides	Seq. cov. (%)
39	133	HBB_HUMAN	Hemoglobin subunit beta	P68871	15.988	6.75	9	74%
40	58	FETUA_HUMAN	Alpha-2-HS-glycoprotein	P02765	39.3	5.43	7	19%
41	112	A1AT_HUMAN	Alpha-1-antitrypsin	P01009	46.707	5.37	11	36%
43	102	ZA2G_HUMAN	Zinc-alpha-2-glycoprotein	P25311	34.237	5.71	14	50%
44	143	ZA2G_HUMAN	Zinc-alpha-2-glycoprotein	P25311	34.237	5.71	18	54%
45	71	HPT_HUMAN	Haptoglobin	P00738	45.177	6.13	12	33%
46	81	HPT_HUMAN	Haptoglobin	P00738	45.177	6.13	12	33%
47	90	HPT_HUMAN	Haptoglobin	P00738	45.177	6.13	13	32%
48	68	ALBU_HUMAN	Serum albumin	P02768	69.321	5.92	14	23%
50	69	ALBU_HUMAN	Serum albumin	P02768	69.321	5.92	15	24%
55	57	OFD1_HUMAN	Oral-facial-digital syndrome 1 protein	O75665	116.599	5.82	14	18%
57	97	TRFE_HUMAN	Serotransferrin	P02787	77.014	6.81	16	18%
58	72	TRFE_HUMAN	Serotransferrin	P02787	77.014	6.81	13	15%
63	58	MIPEP_HUMAN	Mitochondrial intermediate peptidase	Q99797	80.589	6.6	13	19%
67	57	CCD73_HUMAN	Coiled-coil domain-containing protein 73	Q6ZRK6	124.077	5.42	15	17%
68	65	CMIP_HUMAN	C-Maf-inducing protein	Q8IY22	82.922	6.54	13	23%
71	102	HBB_HUMAN	Hemoglobin subunit beta	P68871	15.988	6.75	9	74%
73	66	CCD73_HUMAN	Coiled-coil domain-containing protein 73	Q6ZRK6	124.077	5.42	16	16%
74	58	DNJC8_HUMAN	DnaJ homolog subfamily C member 8	O75937	29.823	9.04	5	29%
75	57	PRDX2_HUMAN	Peroxiredoxin-2	P32119	21.878	5.66	6	38%
77	60	MACF4_HUMAN	Microtubule-actin cross-linking factor 1, isoform 4	Q96PK2	669.721	5.2	24	6%
78	76	P2R3C_HUMAN	Serine/threonine-protein phosphatase 2A regulatory subunit B′′ subunit gamma	Q969Q6	53.282	5.07	13	23%
79	105	TTHY_HUMAN	Transthyretin	P02766	15.877	5.52	7	59%
80	82	TTHY_HUMAN	Transthyretin	P02766	15.877	5.52	6	55%
84	62	ZN774_HUMAN	Zinc finger protein 774	Q6NX45	55.032	8.71	7	20%

## Abbreviations

2DE: 2-dimensional electrophoresis
BSA: Bovine serum albumin
IPGs: Immobilized pH gradients
IEF: Isoelectric focusing
kDa: Kilodaltons
*μ*g: Microgram
*μ*L: Microliter
mg: Milligram
mL: Milliliter
nm: Nanometer
SDSPAGE: Sodium dodecyl sulfate polyacrylamide gel electrophoresis.
